# Thomas Ligertwood

**Published:** 1911-05-20

**Authors:** 


On May 10, at 16 Tedworth Gardens, S.W., Thomas Ligertwood, C.B., K.L.H., M.D., R.A.M.C., late physician and surgeon to the Royal Hospital, Chelsea, aged eighty-one.

